# Ultrasensitive nonlinear absorption response of large-size topological insulator and application in low-threshold bulk pulsed lasers

**DOI:** 10.1038/srep14856

**Published:** 2015-10-07

**Authors:** Jin-Long Xu, Yi-Jian Sun, Jing-Liang He, Yan Wang, Zhao-Jie Zhu, Zhen-Yu You, Jian-Fu Li, Mitch M. C. Chou, Chao-Kuei Lee, Chao-Yang Tu

**Affiliations:** 1 Key Laboratory of Optoelectronic Materials Chemistry and Physics of CAS, Fujian Institute of Research on the Structure of Matter, Chinese Academic of Sciences, Fuzhou, 350002 China; 2 State Key Laboratory of Crystal Materials, Shandong University, Jinan, 250100, China; 3 Department of Materials and Optoelectronics Science, National Sun Yat-sen University, 70, Lienhei Road, Kaohsiung, Taiwan; 4 Department of Photonics, National Sun Yat-sen University, 70, Lienhei Road, Kaohsiung, Taiwan

## Abstract

Dirac-like topological insulators have attracted strong interest in optoelectronic application because of their unusual and startling properties. Here we report for the first time that the pure topological insulator Bi_2_Te_3_ exhibited a naturally ultrasensitive nonlinear absorption response to photoexcitation. The Bi_2_Te_3_ sheets with lateral size up to a few micrometers showed extremely low saturation absorption intensities of only 1.1 W/cm^2^ at 1.0 and 1.3 μm, respectively. Benefiting from this sensitive response, a Q-switching pulsed laser was achieved in a 1.0 μm Nd:YVO_4_ laser where the threshold absorbed pump power was only 31 mW. This is the lowest threshold in Q-switched solid-state bulk lasers to the best of our knowledge. A pulse duration of 97 ns was observed with an average power of 26.1 mW. A Q-switched laser at 1.3 μm was also realized with a pulse duration as short as 93 ns. Moreover, the mode locking operation was demonstrated. These results strongly exhibit that Bi_2_Te_3_ is a promising optical device for constructing broadband, miniature and integrated high-energy pulsed laser systems with low power consumption. Our work clearly points out a significantly potential avenue for the development of two-dimensional-material-based broadband ultrasensitive photodetector and other optoelectronic devices.

Passive Q-switching and mode-locking of lasers are the essential techniques for generating giant laser pulses with pulse width ranging from microseconds to femtoseconds. In such a pulsed laser, the saturable absorber is the key component, as it absorbs the light at low intensity, and becomes transparent at high intensity. The most well-known mature saturable absorber is the semiconductor saturable absorber mirror (SESAM)^1^,^2^,^3^,^4^,^5^,^6^,^7^. The SESAM, however, has several drawbacks, such as narrow spectral operation band within tens of nanometers, complex epitaxial growth fabrication, and an expensive packaging technique. These problems strongly limit its application and thus restrict the present development of pulsed lasers. In recent years, graphene, a two-dimensional hexagonally arrayed carbon atomic material with zero bandgap at the Dirac point, has been confirmed as having the outstanding advantages of broadband absorption, controllable modulation depth, high damage threshold, and low nonsaturable loss^8^,^9^,^10^. These make graphene rather favorable for efficient Q-switching and mode locking of various fiber and bulk lasers in the wavelength range extending from near IR to middle IR^9^,^10^,^11^,^12^,^13^,^14^,^15^,^16^,^17^. We also presented the excellent performance of graphene in bulk pulsed lasers in our previous works^18^,^19^,^20^,^21^,^22^. These encouraging results have aroused considerable interest in finding out whether some other materials, especially the Dirac materials, have the strikingly optical and saturable absorption properties comparable with graphene. The results of the current active research suggest that topological insulators are the most possible candidates.

Topological insulators in two (2D) and three (3D) dimensions are now a focus theme in condensed-matter physics due to their unique phases of quantum matter, which are related closely to the quantum spin Hall effect originating from spin-orbit interactions^23^,^24^,^25^,^26^,^27^,^28^,^29^,^30^,^31^. Just like ordinary insulators, topological insulators have insulating bulk energy gaps separating the conduction and valence bands. However, their edges or surfaces are gapless, leading to a particular conducting metallic state that is protected by time-reversal symmetry. The 2D topological insulator phase has been confirmed in HgTe/CdTe sandwich quantum wells^23^. When the thickness of HgTe exceeds the critical value of ~6.3 nm, the quantum phase transits from a trivial insulator to a quantum spin Hall insulator with the appearance of a pair of gapless helical edge states^23^,^25^. The first experimentally certified 3D topological insulator is the semiconducting alloy Bi_1–x_Sb_x_ (with x = 0.07~0.22), whereas it has a very complex topological surface state that is not easily describable^32^. The simplest topological surface phase, which only consists of a single Dirac cone near the Г point of the surface Brillouin zone, has been recently observed in a class of layered stoichiometric compounds, Bi_2_Se_3_, Bi_2_Te_3_ and Sb_2_Te_3_^29^,^33^,^34^. Within these materials, the heavy elements induce strong spin-orbit coupling with energy larger than the bulk bandgap and, consequently, the coupling effect can modify the surface state to the metallic phase. Such a simple electronic state can be well described, allowing better exploration of the topological states, such as modulation of the energy gap by magnetic impurities and disorders^35^,^36^, the topological magnetoelectric effect^37^, as well as the quantum anomalous Hall effect^38^. In particular, the gapless surface states where the Dirac electrons are linearly dispersive, and the narrow bulk gaps (0.3 eV for Bi_2_Se_3_^29^, 0.162 eV for Bi_2_Te_3_^39^, and 0.28 eV for Sb_2_Te_3_^40^) offer the topological insulators the promising ability of photo-absorption over a broad spectral range. In 2012, Bernand and Zhang first verified the saturable absorption effect of Bi_2_Te_3_ by measuring its nonlinear transmittance at 1.5 μm^41^. Soon after, mode locking operations of 1.5 μm Er fiber lasers and 1.9 μm Tm-Ho fiber laser were successfully realized using Bi_2_Se_3_, Sb_2_Te_3_, and Bi_2_Te_3_^42^,^43^,^44^,^45^,^46^,^47^,^48^. Q-switching has also been achieved in a Yb fiber laser with 1.95 μs pulses^49^, in Er fiber with 13 μs pulses^50^, and in Tm fiber with 4.18 μs pulses^51^ based on topological insulators. Compared with fiber lasers, solid-state bulk lasers have the advantages of compact volume, large mode area, high thermal conductivity and a low undesirable nonlinear effect, making them more suitable for production of high-energy short pulses. In 2013, Tan *et. al.* reported the first study of the performance of topological insulators absorber based solid state pulse laser. in 1.6 μm Bi_2_Te_3_-based Q-switched Er:YAG solid-state lasers^52^. Laterly, a 1.0 μm Bi_2_Se_3_-based Q-switched Nd:GdVO_4_ laser has been also demonstrated by Zhang’s group^53^. A Bi_2_Se_3_ based Q-switched Nd:LiYF_4_ nanosecond laser at 1313 nm is reported recently^54^. However, the obtained pulse durations were 6.3 μs, 666 ns and 433 ns, respectively, still longer than the bulk lasers Q-switched with common SESAMs and doped crystals. Furthermore, we note that graphene has been reported as exhibiting ultrasensitive photoresponsivity in photodetection^55^,^56^. We therefore wonder whether, similar to other Dirac materials, the topological insulators have the similar sensitivity to photoexcitation, and whether it can be exploited to optimize the pulsed laser performance. In this paper, we demonstrated the sensitive saturable absorption of large-size Bi_2_Te_3_ sheets. Taking advantage of these sheets, a low-threshold Q-switched Nd:YVO_4_ laser was achieved at 1.0 and 1.3 μm. The threshold absorbed pump power for the 1.0 μm Q-switched laser was only 31 mW, which is lower than other known low-threshold passively Q-switched bulk lasers^3^,^21^,^57^,^58^,^59^,^60^,^61^. Pulse durations of 97 and 93 ns were obtained in this Bi_2_Te_3_ Q-switched laser at 1.0 and 1.3 μm, respectively. The Q-switched mode locking operation was also obtained. These results indicate that the topological insulator Bi_2_Te_3_ is a promising saturable absorber for low-threshold pulsed lasers. Such lasers have the potential application of efficiently producing high-energy short pulses in miniature and integrated laser systems without high pump intensity.

## Results

### Preparation of topological insulator samples

**Figure 1:**
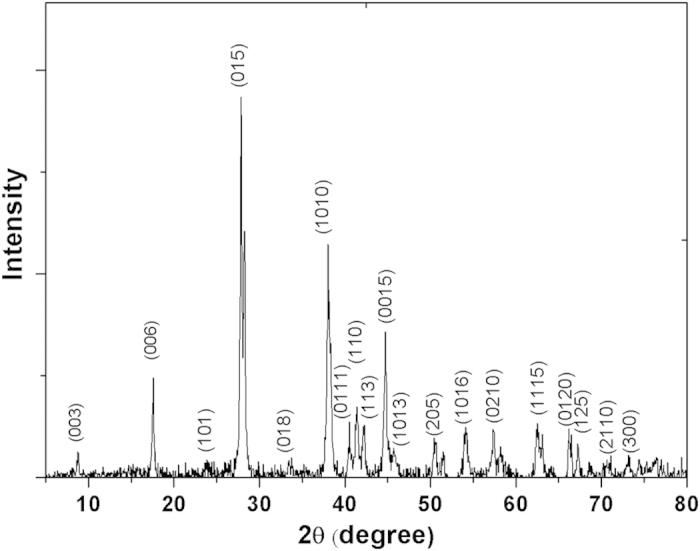
XRD of the Bi_2_Te_3_ powders.

**Figure 2:**
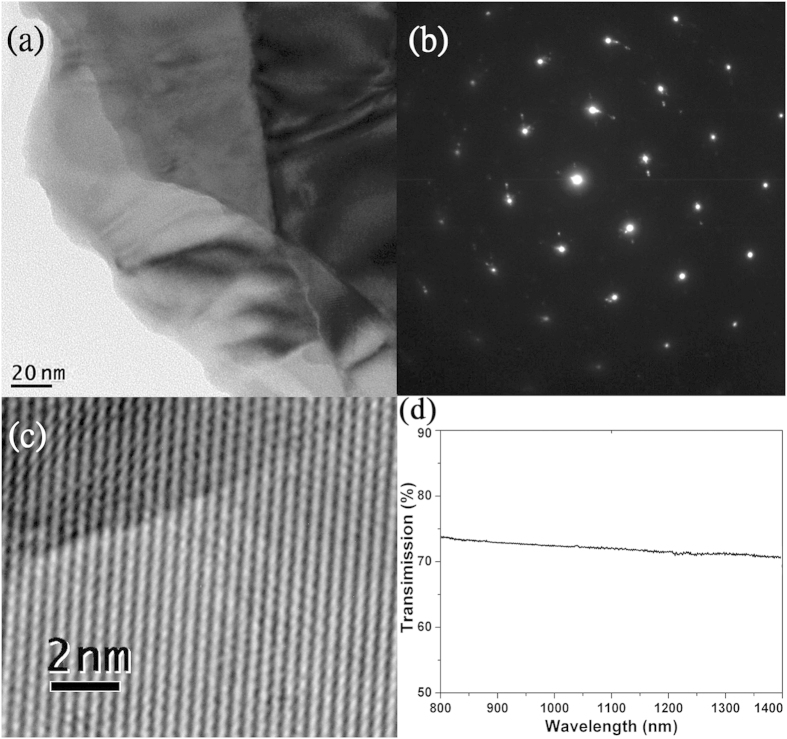
(**a**) TEM image of as-prepared Bi_2_Te_3_ sheets. (**b**) Corresponding SAED pattern. (**c**) HRTEM image showing the crystalline structure. (**d**) Linear absorption spectrum of the Bi_2_Te_3_ dispersion.

We prepared Bi_2_Te_3_ powders by mechanically exfoliating the commercial Bi_2_Te_3_ crystal used for thermoelectricity. Figure 1 presents the X-ray diffraction (XRD) pattern of these powders. The diffraction peaks are well in accordance with the characteristic peaks of rhombohedral Bi_2_Te_3_, identifying these powders as single phase with a space group 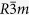. To obtain the Bi_2_Te_3_ sheets, we dispersed the powders in ethanol, followed by ultrasonication for 8 hours. The concentration of Bi_2_Te_3_ in the dispersion was 3 mg/cm^3^. The dispersion was deposited onto a bronze grating substrate and then inspected by transmission electron microscopy (TEM). A large number of Bi_2_Te_3_ sheets with a lateral size ranging from hundreds of nanometers up to several micrometers were observed. The thickness was determined to be 10 ~ 60 nm for different sheets. Figure 2a exhibits the edges of some large-size sheets of about 1.1 ~ 1.3 μm. One can see that the Bi_2_Te_3_ sheets have a quasi-2D layered structure. The corresponding selected area electron diffraction (SAED) shown in Fig. 2b indicates single crystalline nature of these sheets. Figure 2c shows the high-resolution transmission electron microscopy (HRTEM) with the resolution down to 0.2 nm. The uniform hexagonal lattice fringes further confirm the crystalline nature of the as-prepared samples. We next measured the linear transmission of the Bi_2_Te_3_ dispersion as shown in Fig. 2d. Owing to the gapless surface and narrow bulk gap of 0.162 eV^39^, the linear absorption works over a wide spectral range. Within the region of 800 to 1400 nm, the transmission decreases slightly from 74% to 71%.

### Characteristics of saturable absorbers

**Figure 3:**
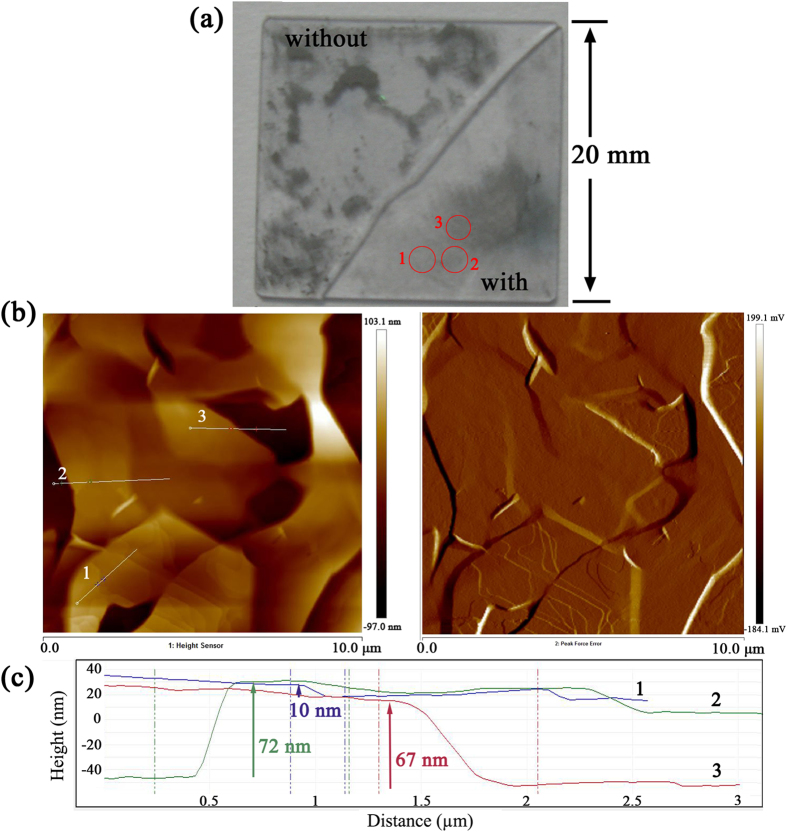
(**a**) Comparison of the distribution of different Bi_2_Te_3_ dispersions (with and without ultrasonic separation) on quartz substrate. The nonlinear transmission measurement was carried on the three circle regions. The film concentration and thickness slightly increase with the number. (**b**) Morphology of the Bi_2_Te_3_ sheets on quartz plate measured by AFM. (**c**) Corresponding height profile diagram of the three positions marked in (**b**).

To apply these sheet samples in laser modulation, we deposited two types of Bi_2_Te_3_ dispersion (with and without 8-hour ultrasonic treatment) onto thin quartz substrates and then dried them in a drying oven for 24 hours. The photograph of the two dried quartz plates is presented in Fig. 3a. The distribution of the sheets with ultrasonication is obviously more uniform, indicating the good separation effect of the ultrasonic treatment for layer-structured materials. AFM measurement (Dimension FastScan, Bruker Cop.) was performed on the dried plate in order to finely estimate the film formation of the evaporated dispersion. It appears that, from the AFM photographs in Fig. 3b, a great deal of Bi_2_Te_3_ sheets folded and overlapped each other, finally composed a large-area film on the substrate surface after evaporation. As in Fig. 3c, the height measurement on three positions shows a thickness range from 10 nm up to 60 nm, coincident with the thickness of Bi_2_Te_3_ sheets verified by TEM. The coverage of the Bi_2_Te_3_ film on the substrate surface was measured as 89%, with a little space left since the uncontrollable free running of the dispersion before complete evaporation.

**Figure 4:**
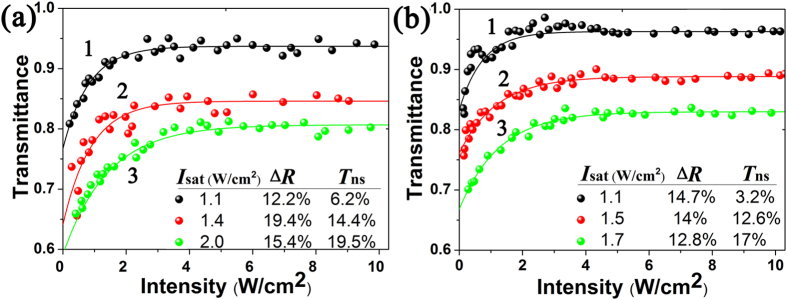
Power-dependent nonlinear transmittance using (**a**) 1064 nm and (**b**) 1342 nm CW lasers and their corresponding fitting curves. The numbers refer to the three circle regions in Fig. 3a.

We measured the nonlinear absorption properties at the three regions (as marked in Fig. 3a) using the pump-probe method with two continuous-wave (CW) lasers at 1.0 and 1.3 μm perpendicularly illuminating the sample. It should be noted that for a bulk laser the diameter of the intracavity fundamental beam on a saturable absorber is usually beyond 100 μm. In purpose of valuing the capability in such a bulk laser, the laser beams in the pump-probe measurement were focused to a radius of 0.4 mm, which is comparable to the size of an intracavity beam. From the measured transmission curves and extracted data (i.e. saturation intensity *I*_sat_, modulation depth ∆*R* and nonlinear transmission *T*_ns_) shown in Fig. 4, one can see that nonlinear saturable absorption response was obtained unambiguously under very weak photoexcitation in all three regions. *I*_sat_ was as low as 1.1 W/cm^2^ both at 1.0 and 1.3 μm, and increased slightly from region 1 to region 3, indicating that the optical response is affected by film thickness and concentration, but they are not the critical elements to induce such an ultrasensitive absorption. Furthermore, the comparison between 1.0 and 1.3 μm excitation shows similar optical properties in each region, related to the slightly wavelength-dependence linear absorption of the Bi_2_Te_3_ dispersion as presented in Fig. 2d. Then we were aware of the sensitive photoresponse of the Bi_2_Te_3_ bulk state which is a combination of direct bandgap and indirect bandgap due to spin-orbit interaction^33^,^62^,^63^ as discussing in the follows.

**Figure 5:**
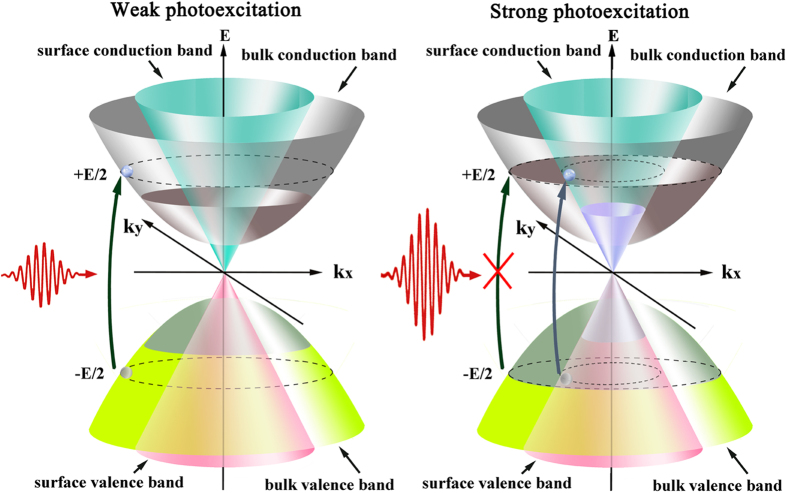
Schematic saturable absorption response dynamics in the bulk and surface states under (**a**) weak and (**b**) strong photoexcitation. (**a**) The photo-absorption in the subsurface bulk region plays a critical role in the saturable absorption dynamic under weak photoexcitation due to the much more sensitive photoresponse than the surface state. The surface state dominates the saturable absorption effect under strong photoexcitation, where the bulk-state absorption remains long full saturation and the electron transition is blocked.

Typically, the reported saturation intensities of topological insulator nanosheets are up to the order of GW/cm^2^ frequently^42^,^43^,^46^,^49^, almost the same to the pure graphene. By comparison, the opto-electronic conversion responsivity of our samples is extraordinarily sensitive. Ultrasensitive photoresponse has been successfully explored in the photodetectors fabricated based on graphene or MoS_2_^55^,^56^,^64^. The photoresponsivity of pure graphene is 10 mAW^−1^ 55 (this unit means photo-absorption-generated current per incident optical power), which is higher than many common semiconductors, but is still limited by its relatively weak optical absorption^65^,^66^,^67^. Soon afterwards, the photosensitivity of graphene was improved to 8.61 AW^−1^ by introducing electron trapping centers and creating a narrow direct bandgap at the Dirac point through band structure engineering, which contributed to slow electron-hole recombination and thus enhanced the light absorption^56^. For MoS_2_, the multi-layer bulk is an indirect-gap semiconductor with a bandgap of 1.2 eV^68^, and the single layer is a direct-gap semiconductor with a bandgap of 1.8 eV^69^, owing to the quantum confinement effect^70^. The recently reported photodetector of pure monolayer MoS_2_ exhibits ultrahigh photosensitivity of 880 AW^−1^, which is attributed to the inherent advantage of its direct bandgap structure^64^, relative to 100 mAW^−1^ for multi-layer MoS_2_^71^. The femtosecond fiber lasers based on MoS_2_ have been also successfully achieved^72^,^73^. These reports prove that a narrow direct bandgap provides quasi-2D layered semiconducting material strong absorption. This make the intriguing narrow-direct-energy-band bulk state of Bi_2_Te_3_ much more sensitive than the gapless surface to photoexcitation, and therefore a very low saturation intensity is achieved. It is worth noting that there is no hybridization between the surface Dirac cone and the bulk band state in the proximity of the Fermi energy E_F_^33^,^34^, so that the processes of carrier generation and recombination transitions between the surface and bulk states after photoexcitation can be discriminated^74^. We present two pictures in Fig. 5 to further illustrate the difference in response dynamic between the bulk and surface states. Strong bulk absorption excites the transition of electrons efficiently from the valence band to conduction band even under very low illumination intensity. The excitation is periodically blocked (i.e., absorption is saturated) at the time that all the available states in conduction band are occupied by photo-generated carriers. On the contrary, for a strong photoexcitation far beyond the saturation intensity, the bulk state reached a stationary saturation condition with equilibrium carrier population, which keeps the bulk long transparent for excited light due to the Pauli blocking. If the photoexcitation intensity is strong enough to be comparable to the surface *I*_sat_, surface saturable absorption will start up in the Dirac cone. The surface and bulk absorptions of course coexist in all Bi_2_Te_3_ sheets, even though the sensitivity and strength differ for different sheet size and surface-to-volume ratio. In the previous pump-probe measurements by other authors^42^,^43^,^45^,^46^,^49^, all the light sources were femtosecond lasers with power intensity of the order MW/cm^2^-GW/cm^2^, at least million times higher than the saturation intensity of bulk state. This fact means that the bulk absorption was absolutely blocked even under the minimum intensity, which should be the reason why the ultrasensitive bulk absorption was not discovered in the previous works. For our samples, bulk physics is stronger than surface physics since the large sheet size is up to micrometer scale with only 10–60 nm in thickness, unlike the nanosheets which have a large surface-to-volume ratio. In this case, the bulk state plays a dominant role in the sensitive saturable absorption dynamic, similar to the reported bulk-structured Bi_2_Te_3_^45^,^52^, but different from the topological insulator nanosheets where the gapless surface state is largely responsible for the saturable absorption effect. The larger the subsurface bulk region, the more sensitive the absorption. This make it possible to achieve selective sensitivity on the saturable absorption response by well controlling the surface-to-volume ratio. But the sheet thickness is just confined to an appropriate extent considering that thinner sheet has a weaker direct-band bulk physics, while thicker one will depress quantum-mechanical confinement and introduce more intrinsic loss that deteriorate its optical property. Strong saturable absorption is also termed large Δ*R*, which reaches 12.2%–19.4% and 12.8%–14.7% for 1.0 and 1.3 μm, respectively, as shown in Fig. 4. Generally it is known that large modulation depth is critical for shortening the pulse duration in both passively Q-switched and mode-locked lasers^75^,^76^. These properties make large-size Bi_2_Te_3_ a favorable device for low-threshold short-pulse laser generation.

### 1.0 μm Q-switched laser with Bi2Te3 saturable absorber

**Figure 6:**
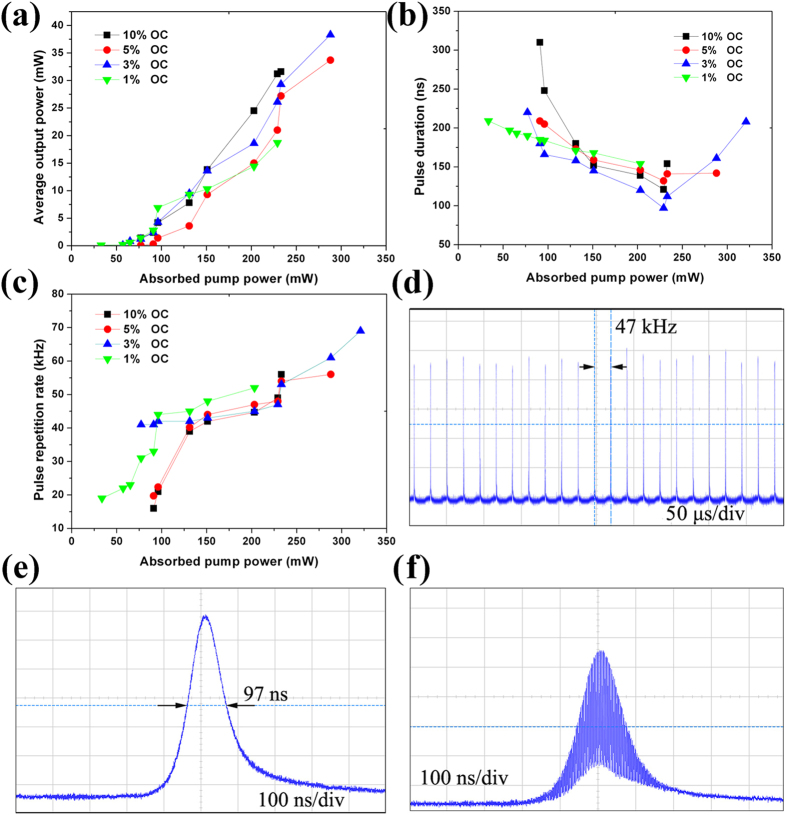
(**a**) Average output power, (**b**) Q-switched pulse duration, and (**c**) repetition rate of the Q-switched laser at 1.0 μm versus absorbed pump power. (**d**) Q-switched pulse train under the average output power of 26.1 mW with 3% OC. (**e**) Corresponding single pulse with a duration of 97 ns. (**f**) Mode-locked pulses within a Q-switched pulse under the average output power of 38.3 mW with 3% OC.

Firstly, we evaluated the performance of the large-size Bi_2_Te_3_ sheets for 1.0 μm Q-switched bulk laser. A detailed description of the experimental laser design is given in the Methods section attached behind. The position and inclination of the Nd:YVO_4_ and cavity mirrors were carefully adjusted to their optimization when the laser was free running. We then switched the pump laser off, and placed the quartz plate coating with the Bi_2_Te_3_ sheets (the right sample in Fig. 3a) close to the OC. Based on the ABCD propagation matrix, the radius of the intracavity fundamental mode on the sheets was calculated to be about 70 μm. It was exciting to find that, by using the four OCs, stable emission of Q-switched pulses was realized under very low pump level, as shown in Fig. 6a. The threshold absorbed pump power was only 31 mW for 1% OC. Further increasing the absorbed pump power resulted in a monotonic rise in average output power. The central wavelength was measured to be 1064 nm. As one can see from Fig. 6b,c, the pulse duration and repetition rate strongly depended on the pump power, which is a typical characteristic of passively Q-switched lasers^76^,^77^,^78^,^79^,^80^, indicating that Bi_2_Te_3_ indeed served as a saturable absorber. For common passively Q-switched lasers with doping crystals as saturable absorbers, such as Cr^4+^:YAG and V^3+^:YAG, noise-like intensity fluctuation usually exists in low output power regions because of the unbleaching of the absorbers, whereas no fluctuation was observed in this Q-switched laser. For all four OCs, the laser operation entered a Q-switched regime immediately when the laser exceeded the threshold, in line with the sensitive saturable absorption effect of these large-size Bi_2_Te_3_ sheets. For instance, in the case of 1% OC, stable yielding of giant pulses with 209 ns duration and 19 kHz repetition rate was detected under the absorbed pump power of 32 mW, even though the average output power was only 0.1 mW. Stable Q-switching should be achieved at lower pump power, but it excesses the sensitivity of our photodetector and power meter. The pulse duration of 97 ns was obtained under 229 mW absorbed pump power by using 3% OC, with an average output power of 26.1 mW and a repetition rate of 47 kHz, giving a pulse energy of 0.6 μJ. The corresponding pulse train and single pulse profile are shown in Fig. 6d,e. To investigate the stability of Q-switching, we further evaluated the quality of pulse–amplitude equalization. This can be characterized by clock amplitude jitter (CAJ) which is defined as the ratio of the standard deviation 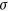 to the mean value (M) of the intensity histogram at the pulse peak intensity, as described below^81^,^82^,^83^


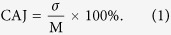


The CAJ of the pulse train in Fig. 6d was calculated as being 4.2%, revealing a good intensity stability.

It is of particular interest to note that absorbed pump power higher than 280 mW introduced noticeable mode locking modulation in the Q-switched laser with 3% OC. The mode-locked pulse train can be clearly seen from Fig. 6f recorded under the absorbed pump power of 288 mW. The corresponding average output power is 38.3 mW. Under mode locking modulation, the Q-switched pulses were broadened (see Fig. 6b), because a large amount of the population inversion was consumed by the mode-locked pulses^84^,^85^. The repetition rate of the mode-locked pulses should be ~5 GHz in accord with the 8-mm-long resonator. However, accurate measurement of the actual values was limited by the 500 MHz bandwidth of our oscilloscope. The transition from pure Q-switching to mode locking was reproducible. Pure continuous-wave mode locking operation was not achieved, possibly attributed to the inhomogeneity of the Bi_2_Te_3_ film. The film is good enough in quality for building a low-threshold Q-switched bulk laser as proved in this paper, but still not enough for a mode-locked bulk laser because its stability is much more sensitive to saturable absorption fluctuation resulting from film inhomogeneity. Whereas a stable mode-locked bulk laser with very low threshold is predictable with further improving the film homogeneity and resonator optimization.

### 1.3 μm Q-switched laser with Bi_2_Te_3_ saturable absorber

**Figure 7:**
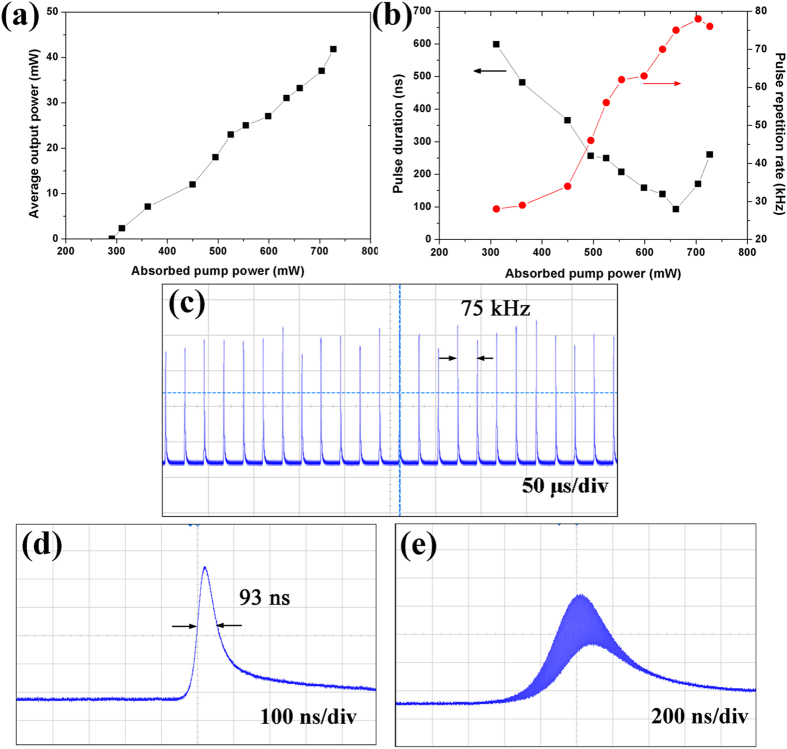
(**a**) Average output power, (**b**) Q-switched pulse duration and repetition rate of the Q-switched laser at 1.3 μm versus absorbed pump power. (**c**) Q-switched pulse train under the average output power of 33.2 mW. (**d**) Corresponding single pulse with duration of 93 ns. (**e**) Mode-locked pulses within a Q-switched pulse under the average output power of 37 mW.

Furthermore, we tested the 1.3 μm pulsed laser performance of the large-size Bi_2_Te_3_ sheets by replacing the 1.0 μm IM and OC with 1.3 μm ones. The resonator structure was the same as that for 1.0 μm laser described in the Methods section. The IM with a curvature radius of 500 mm was antireflection coated at 808 nm and high-reflection coated at 1300–1360 nm. The OC was a plane mirror with 4% transmission at 1300–1360 nm. The intracavity fundamental mode on the sheets had a radius of about 70 μm according to the laser configuration. Similar to the aforementioned 1064 nm case, the 1342 nm Q-switching initiated as the laser oscillation started under the threshold absorbed pump power of 291 mW. Compared with the above 1064 nm case, the relatively high threshold should be due to the smaller stimulated emission section of Nd:YVO_4_ at 1342 nm. However, the threshold is rather lower than some reported 1.3 μm low-threshold passively Q-switched bulk lasers, e.g., 0.55 W threshold pump power for V^3+^:YAG Q-switched Nd:YVO_4_ laser^61^, and 0.63 W for graphene Q-switched Nd:GdVO_4_ laser^21^. This result approaches very near to the 250 mW threshold pump power of the 1.3 μm Q-switched Nd:YVO_4_ microchip laser with mature SESAM^3^. A steady increase in average output power with absorbed power can be observed in Fig. 7a. The dependence of the pulse duration and repetition rate on the pump level is presented in Fig. 7b. Under the absorbed pump power of 657 mW, we achieved a pulse duration of 93 ns at 33.2 mW average output power and 75 kHz repetition rate. Figure 7c,d show the corresponding profiles of the Q-switched pulse train and single pulse, respectively. The CAJ of the pulses in Fig. 7c was calculated to be 5.7%, which is close to the 1.0 μm case. This is the shortest pulse duration among the reported topological-insulator-based passively Q-switched bulk and fiber lasers^49^,^50^,^51^,^52^,^53^,^54^. We also observed the building up of mode-locked pulse train as the absorbed pump power was higher than 657 mW, as shown in Fig. 7e. The mode-locking modulation realized at both 1.0 and 1.3 μm reflects the capability of Bi_2_Te_3_ in wideband mode-locked bulk lasers. The chemical properties of these Bi_2_Te_3_ sheets are so stable that the Q-switching experiment was reproducible a week later. We did not observe any damage to the Bi_2_Te_3_ sample during the Q-switching experiment. The damage threshold was measured to be around 100 MW/cm^2^ by using a mode-locked Ti:sapphire laser with 100 fs pulse duration, exhibiting good thermal stability against optical damage.

## Discussion

The distance from the focal point of the pumping laser to the Bi_2_Te_3_ film was ~6 mm. We used a pyroelectric camera (Pyrocam III, Ophir Optronics Ltd.) to measure the spatial profiles of the multimode pumping beam, and found that the beam diverged to a radius of 3.1 mm at the position of the Bi_2_Te_3_ film. For the stable Q-switched laser we achieved under 32 mW absorbed pump power, the remaining pump power was just 3.5 mW, giving a residual power intensity of 0.01 W/cm^2^ that is much lower than the minimum saturation absorption intensity of 1.0 W/cm^2^. Even under the maximum absorption power of 724 mW in the experiment, corresponding to a remaining power of 65 mW, the intensity on the Bi_2_Te_3_ was only 0.22 W/cm^2^, still ~5 times lower than the measured minimum saturation absorption intensity. Therefore, we believe that the influence of the residual pump power on the saturable absorption procedure of Bi_2_Te_3_ can be ignored.

In conclusion, large-size Bi_2_Te_3_ sheets offered ultrasensitive saturable absorption response to photoexcitation, which should be mainly attributed to the strong bulk state of the narrow bandgap in the large sheets. With these Bi_2_Te_3_ sheets as saturable absorber, a low-threshold passively Q-switched Nd:YVO_4_ laser has been achieved at both 1.0 and 1.3 μm. Bi_2_Te_3_-induced mode locking in the Nd:YVO_4_ laser was also observed. By using large-size Bi_2_Te_3_ and with further resonator optimization, low-threshold continuous-wave mode-locked bulk lasers are foreseeable. One can also expect that selective sensitivity on the absorption response is achievable by well controlling the surface-to-volume ratio. Therefore, large-size Bi_2_Te_3_ sheets may be of particular interest for optoelectronic applications in low-pumping high-energy miniature and integrated pulsed laser systems.

## Methods

### Low-threshold Q-switched laser design

The carrier relaxation dynamics of Bi_2_Te_3_ are very fast after photoexcitation. The thermalization of interband scattering lasts for about several hundred femtoseconds, and the following recovery of a Fermi-Dirac distribution is of the order of 10 ps^74^. Then the Bi_2_Te_3_ behaves as a fast Q-switcher. Based on ref. 86, 87, the threshold condition for passively Q-switched laser with Bi_2_Te_3_ as a fast saturable absorber can be derived from


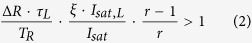


where, 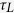 and 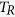 are the upper-state lifetime of the gain medium and the resonator round-trip time, respectively. 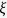 is the factor describing the intensity enhancement resulting from the reflectivity of the saturable absorber. Its value is close to 1 for these high-transmission Bi_2_Te_3_ sheets. *r* is the pump level parameter determining how many times above the threshold the laser is operated. 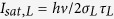 is the saturation intensity of the gain medium with 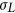 being the gain cross section. Here, the fact of 2 takes into account the influence of the standing wave within the gain medium^57^. From Eq. (2) one can come to the conclusion that the utilization of a saturable absorber with significantly low saturation intensity and large modulation depth, short resonator length, together with a gain medium of long upper-state lifetime and high gain cross section, can construct a Q-switched laser under a very low pump level. We therefore chose a Nd:YVO_4_ crystal as the laser medium for its well-known physical and optical properties, i.e., 98 μs of upper-state lifetime, 25 × 10^−19^ cm^2^ and 13 × 10^−19^ cm^2^ of gain cross sections for 1064 and 1342 nm emissions, respectively^88^.

**Figure 8:**
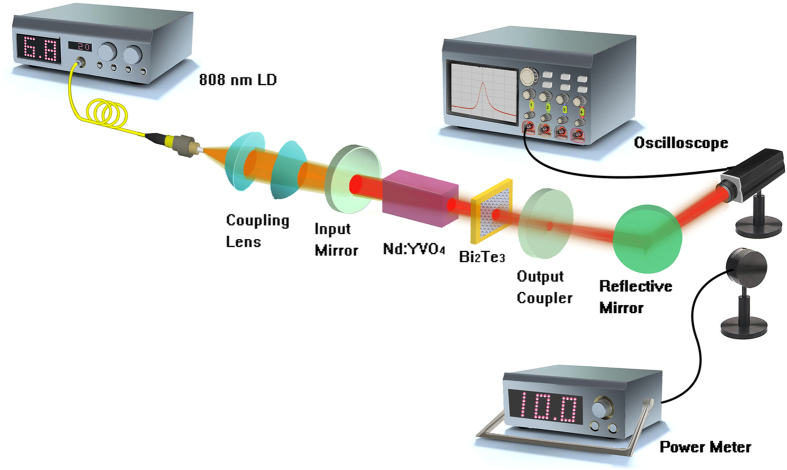
Setup of the passively Q-switched Nd:YVO_4_ laser at 1.0 and 1.3 μm with Bi_2_Te_3_ as saturable absorber.

The Nd:YVO_4_ crystal had a length of 7 mm and a Nd^3+^-ion doping concentration of 0.4%. It was wrapped by indium foil and mounted in a copper block kept at 20 °C to remove the stored heat. The laser setup is schematically presented in Fig. 8. The pump source was a fiber coupled continuous-wave diode laser at 808 nm with a core diameter of 200 μm and a numerical aperture of 0.22. The pump laser was focused to a circular spot of ~100 μm in diameter inside the gain crystal. The absorption efficiency of the Nd:YVO_4_ crystal to the pump power was measured as 92%. For 1.0 μm laser, the input mirror (IM) with a curvature radius of 500 mm was antireflection coated at 808 nm and high-reflection coated at 1040–1080 nm. Four plane output couplers (OCs) with different transmittances of 1%, 3%, 5% and 10% at 1010–1080 nm were utilized to compare the influence on the laser performance. The cavity was compressed to the minimum length of 9 mm. For 1.3 μm laser, the IM was a concave mirror with a curvature radius of 500 mm and antireflection coated at 808 nm and high-reflection coated at 1300–1360 nm. The OC was a plane mirror with 4% transmission at 1300–1360 nm. The intracavity fundamental mode on the Bi_2_Te_3_ film had a radius of 70 μm according to the laser configuration. The laser waveform was detected by a digital oscilloscope (500 MHz bandwidth, 4 Gs/s sampling rate).

## Additional Information

**How to cite this article**: Xu, J.-L. *et al.* Ultrasensitive nonlinear absorption response of large-size topological insulator and application in low-threshold bulk pulsed lasers. *Sci. Rep.* **5**, 14856; doi: 10.1038/srep14856 (2015).
